# Supplementary material for: Genome-Wide Identification of the Litchi BBX Gene Family and Analysis of Its Potential Role in Pericarp Coloring
Source: Int J Mol Sci. 2025 Nov 7;26(22):10834. doi: 10.3390/ijms262210834 (PMC12652159; doi:10.3390/ijms262210834)
Supplement: Supplementary file 1 [file ijms-26-10834-s001.zip › figure legends.pdf]

Figure S1: Motif analysis of *BBX* gene family members in litchi.

Figure S2: Analysis of *Cis*-acting elements in the promoter sequences of *LcBBX* genes.

Figure S3: GO enrichment annotation of the *LcBBX* genes.
