# Supplementary figures and images for: Genome-Wide Identification of the Litchi BBX Gene Family and Analysis of Its Potential Role in Pericarp Coloring
Source: Int J Mol Sci. 2025 Nov 7;26(22):10834. doi: 10.3390/ijms262210834 (PMC12652159; doi:10.3390/ijms262210834)

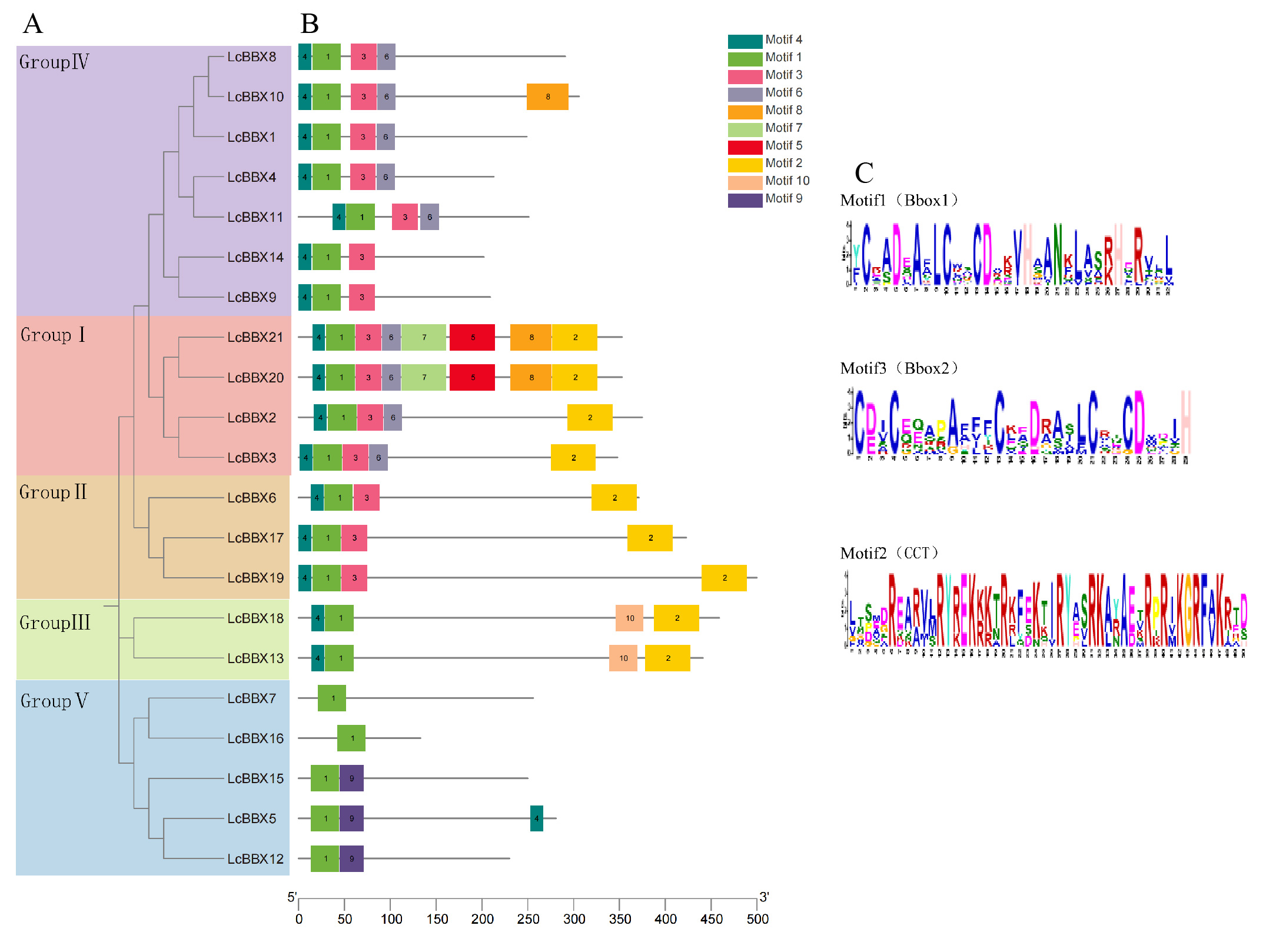

Supplement: Supplementary file 1 [file ijms-26-10834-s001.zip › Figure S1.jpg]

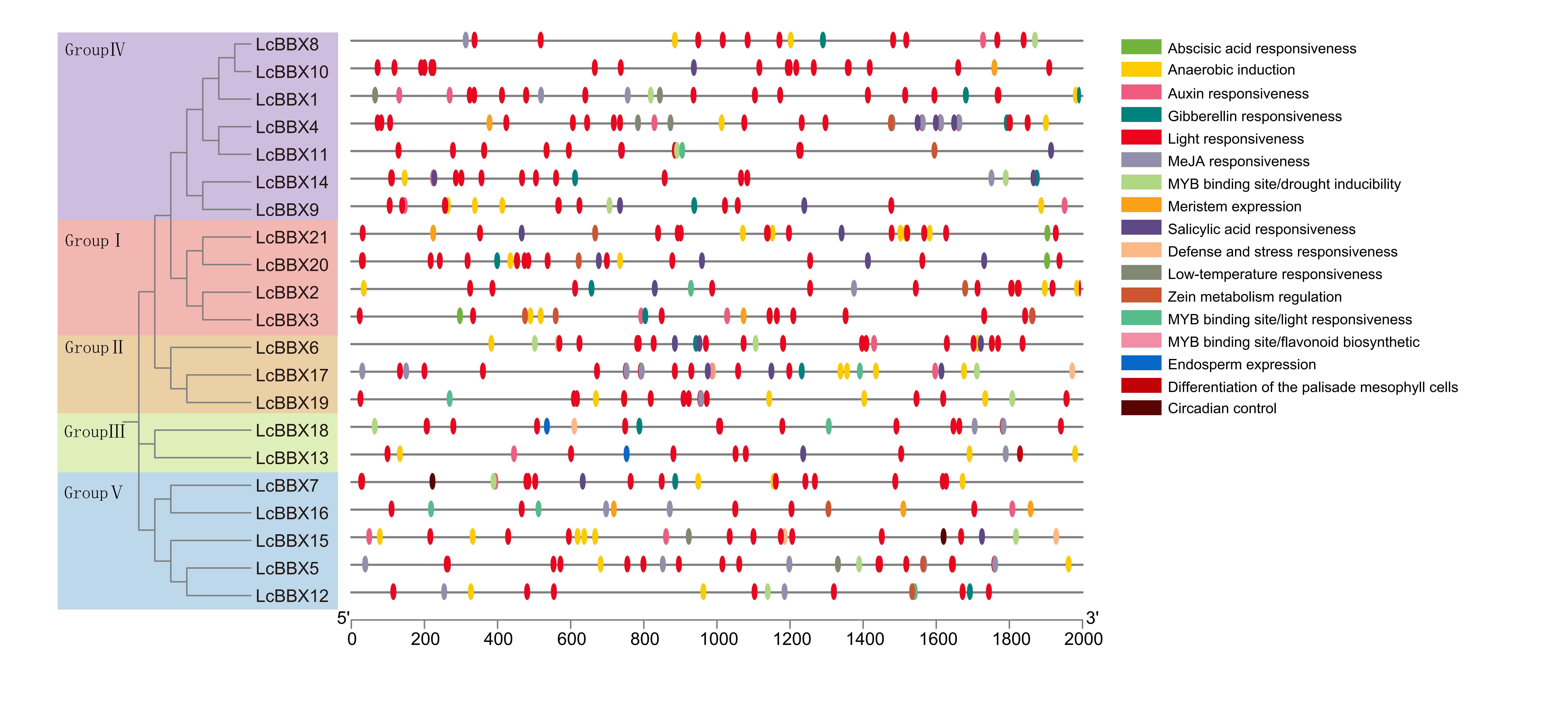

Supplement: Supplementary file 1 [file ijms-26-10834-s001.zip › Figure S2.jpg]

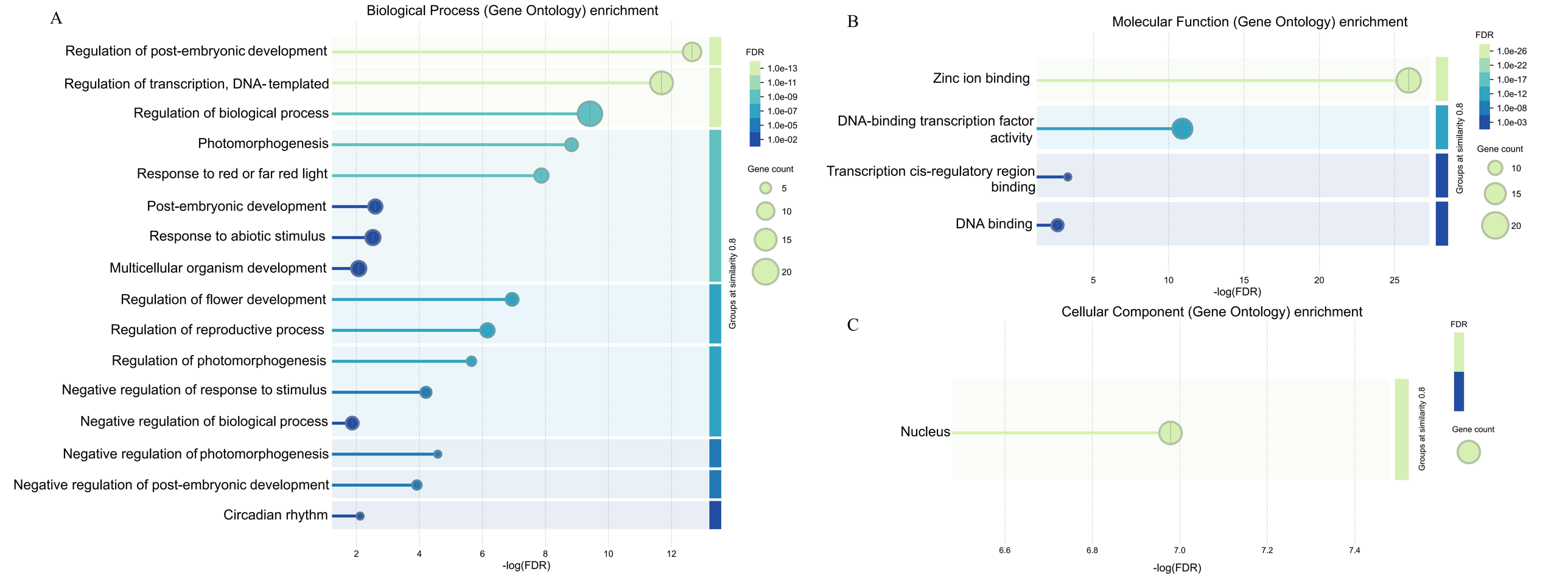

Supplement: Supplementary file 1 [file ijms-26-10834-s001.zip › Figure S3.jpg]
